# Mitochondrial Injury Accompanied by Intermediate Filament Remodeling Following Lithium Chloride Exposure in 3D Endometrial Cancer Spheroids

**DOI:** 10.3390/biomedicines14030655

**Published:** 2026-03-13

**Authors:** Berna Yıldırım, Burcu Biltekin, Mete Hakan Karalök, Ayhan Bilir

**Affiliations:** 1 Department of Histology and Embryology, Faculty of Medicine, İstanbul Atlas University, İstanbul 34408, Turkey; berna.yildirim@atlas.edu.tr (B.Y.); burcu.biltekin@atlas.edu.tr (B.B.); 2 Department of Obstetrics and Gynecology, Faculty of Medicine, İstanbul Atlas University, İstanbul 34408, Turkey; hakan.karalok@atlas.edu.tr

**Keywords:** endometrial cancer, 3D spheroids, mitochondrial stress, intermediate filaments, non-apoptotic stress response, transmission electron microscopy

## Abstract

**Background/Objectives**: Endometrial cancer frequently develops resistance to therapy, partly due to the ability of tumor cells to adapt to cellular stress through non-apoptotic mechanisms. Mitochondrial dysfunction and cytoskeletal remodeling are increasingly recognized as key components of stress adaptation; however, their structural relationship under pharmacological stress in three-dimensional (3D) tumor models remains poorly characterized. The present study aimed to investigate the ultrastructural and phenotypic effects of lithium chloride (LiCl)-induced stress in 3D endometrial cancer spheroids, with a particular focus on mitochondrial alterations and intermediate filament organization. **Methods**: Three-dimensional spheroids generated from Ishikawa endometrial cancer cells were exposed to lithium chloride at concentrations of 1, 10, or 50 mM for defined time periods. Cell viability, proliferative activity, and clonogenic capacity were assessed using Trypan Blue exclusion, BrdU incorporation, and soft agar assays. Ultrastructural changes were examined by transmission electron microscopy to evaluate mitochondrial morphology, cytoplasmic organization, and intermediate filament distribution. **Results**: LiCl exposure resulted in a dose- and time-dependent reduction in cell viability, proliferation, and clonogenic potential in 3D spheroids. Ultrastructural analysis revealed pronounced mitochondrial swelling, cristae disorganization, and membrane-associated mitochondrial alterations. These changes were consistently accompanied by conspicuous accumulation and reorganization of intermediate filaments in close spatial proximity to damaged mitochondria, suggesting a structural association between cytoskeletal remodeling and mitochondrial injury. Across all experimental conditions, classical apoptotic ultrastructural features, including chromatin condensation and apoptotic body formation, were not observed. **Conclusions**: Together, these observations indicate that lithium chloride elicits a stress phenotype in 3D endometrial cancer spheroids that primarily manifests at the organelle and cytoskeletal levels, rather than through classical apoptotic execution. Although descriptive in nature, the present study highlights intermediate filament accumulation as a prominent structural feature of lithium-induced mitochondrial stress and establishes a structural reference point for future studies aimed at further investigating mitochondrial–cytoskeletal relationships during pharmacological stress in endometrial cancer.

## 1. Introduction

Endometrial cancer represents one of the most frequently diagnosed gynecologic malignancies, and although many cases are detected at an early stage, disease recurrence and resistance to therapy continue to pose significant clinical challenges [1,2]. These challenges are not driven solely by genetic alterations but are also shaped by profound changes in cellular organization, including dysregulated cell-cycle control, altered cytoskeletal architecture, and disrupted mitochondrial homeostasis. Together, these alterations critically influence tumor behavior and treatment responsiveness [2,3].

Uncontrolled proliferation is a defining feature of malignant cells [4]. In normal tissues, cell division is tightly regulated by growth factor-dependent signaling and checkpoint mechanisms, whereas cancer cells bypass these controls through aberrant receptor activation and downstream survival pathways [4,5]. Mitochondria play a central role in this adaptation. Beyond their bioenergetic function, mitochondria regulate reactive oxygen species production, calcium signaling, and stress-responsive pathways that directly influence proliferation, differentiation, and cell fate decisions [6,7]. Preservation of mitochondrial integrity therefore depends on coordinated quality-control mechanisms, including organelle turnover and adaptive stress responses [8,9].

In parallel with mitochondrial adaptations, the cytoskeleton, particularly the intermediate filament (IF) network, has emerged as a critical regulator of tumor cell behavior. Intermediate filaments are no longer viewed as static structural components; rather, they actively participate in signal transduction, mechanical resilience, intracellular organization, and cellular stress adaptation [10,11,12,13]. Aberrant expression and reorganization of keratins, vimentin, and nestin have been associated with tumor progression, invasiveness, and therapy resistance in multiple cancer types, including endometrial carcinoma [10,11,12,13,14,15]. Despite this growing recognition, the contribution of intermediate filaments to stress-induced organelle remodeling remains incompletely understood.

Lithium chloride (LiCl), a classical inhibitor of glycogen synthase kinase-3β (GSK-3β) [16], has been reported to exert antiproliferative and cytotoxic effects in a variety of tumor models. These effects are frequently accompanied by mitochondrial stress, redox imbalance, and perturbation of cell-cycle regulators [17,18,19]. However, substantial heterogeneity exists across studies with respect to LiCl concentration, exposure duration, and cellular context, complicating the distinction between adaptive stress responses and overt cytotoxicity [19]. Moreover, how lithium-induced mitochondrial stress intersects with cytoskeletal remodeling processes, particularly within three-dimensional tumor architectures, remains largely unexplored.

In this context, GSK-3β signaling has been increasingly implicated in endometrial carcinoma biology, particularly through its regulatory role in Wnt/β-catenin signaling and cell proliferation pathways [20]. Dysregulation of GSK-3β activity has been reported to influence tumor growth, metabolic adaptation, and resistance to therapeutic stress in several cancer types, including gynecologic malignancies [21]. Although lithium is widely used experimentally as a pharmacological inhibitor of GSK-3β, the downstream structural consequences of lithium exposure in three-dimensional endometrial cancer models remain insufficiently characterized [22].

Three-dimensional (3D) spheroid models provide an experimentally advantageous platform for addressing these questions, as they more closely recapitulate in vivo tumor features such as nutrient and oxygen gradients, cell–cell interactions, and differential drug penetration compared with conventional monolayer cultures [23,24]. Within this context, proliferative dynamics, mitochondrial integrity, and cytoskeletal organization can be examined under conditions that more closely reflect the structural complexity of solid tumors.

Although mitochondrial stress responses and quality-control mechanisms have been widely explored in cancer biology [7,9,25,26], the role of intermediate filament networks in shaping cellular responses to therapeutic or pharmacological stress remains poorly defined. In particular, the spatial and functional relationship between mitochondrial injury and intermediate filament remodeling under stress conditions has received limited attention. To date, no studies have directly examined whether LiCl exposure promotes intermediate filament accumulation or spatially associated mitochondrial injury and cytoskeletal remodeling in endometrial cancer spheroids. Given the increasing recognition of IF proteins as both biomarkers and functional regulators of tumor behavior [27,28,29,30,31,32], addressing this gap is of particular relevance.

Accordingly, the present study investigates the dose- and time-dependent effects of lithium chloride on proliferation, mitochondrial morphology, and intermediate filament organization in 3D Ishikawa endometrial cancer spheroids. By integrating viability and proliferation assays with detailed ultrastructural analysis, we aimed to characterize organelle-centered stress responses and cytoskeletal remodeling associated with lithium exposure, without presupposing specific cell-death mechanisms. Our findings reveal that lithium-induced mitochondrial injury is consistently accompanied by prominent intermediate filament remodeling, providing a descriptive structural framework for future mechanistic studies of mitochondrial–cytoskeletal interactions during stress adaptation in endometrial cancer.

## 2. Materials and Methods

### 2.1. Cell Culture and Lithium Chloride Exposure

The human endometrial adenocarcinoma cell line Ishikawa (ATCC^®^ CRL-12051™, RRID:CVCL_2529) was maintained in RPMI-1640 medium (Gibco, Thermo Fisher Scientific, Waltham, MA, USA) supplemented with 10% fetal bovine serum (FBS; Gibco, Thermo Fisher Scientific, Waltham, MA, USA) and 1% penicillin–streptomycin (Gibco, Thermo Fisher Scientific, Waltham, MA, USA) under standard culture conditions (37 °C, 5% CO_2_, humidified atmosphere). Cells were routinely passaged at approximately 80% confluence using 0.25% trypsin–EDTA (Gibco, Thermo Fisher Scientific, Waltham, MA, USA).

Lithium chloride (LiCl) was freshly dissolved in sterile distilled water and added to the culture medium at final concentrations of 1, 10, or 50 mM. These concentrations were selected based on previous in vitro studies reporting lithium-induced mitochondrial stress and autophagy-associated cellular responses in cancer models [17,18,33]. Control cultures received vehicle alone. Although these concentrations exceed physiological serum lithium levels, short-term exposure to high LiCl doses is widely employed in vitro to interrogate mitochondrial and cytoskeletal stress phenotypes under controlled experimental conditions [17].

### 2.2. Trypan Blue Exclusion Assay

Cell viability was assessed by Trypan Blue exclusion. Following LiCl treatment, cells were collected, mixed at a 1:1 ratio with 0.4% Trypan Blue solution (Sigma-Aldrich, St. Louis, MO, USA), and counted manually using a Neubauer hemocytometer (Marienfeld, Lauda-Königshofen, Germany) under light microscopy. For each replicate, four randomly selected microscopic fields were evaluated, typically comprising 200–400 cells per field. Cells excluding the dye were classified as viable, whereas blue-stained cells were considered non-viable. Experiments were performed using three independent biological replicates, each analyzed in technical duplicate.

### 2.3. Three-Dimensional Spheroid Generation

Multicellular spheroids were generated using a liquid-overlay technique. Six-well culture plates were coated with 3% Noble agar (Sigma-Aldrich, St. Louis, MO, USA), and 1 × 10^6^ viable Ishikawa cells were seeded per well. Plates were incubated for 5–7 days to allow spheroid formation. Spheroids exhibiting uniform spherical morphology, intact architecture, and absence of central necrosis, as confirmed by phase-contrast microscopy, were selected for subsequent experiments. Spheroid diameters ranged between 120 and 300 µm. Selected spheroids were treated with LiCl at concentrations of 1, 10, or 50 mM for 24 or 72 h.

### 2.4. BrdU Proliferation Analysis in Spheroids

Proliferative activity within spheroids was assessed by bromodeoxyuridine (BrdU) incorporation. At the end of treatment, spheroids were fixed in neutral-buffered formalin, routinely processed, and embedded in paraffin. Sections (5 µm) were subjected to antigen retrieval in citrate buffer (pH 6.0) and incubated with an anti-BrdU antibody (Abcam, Cambridge, UK). Immunoreactivity was visualized using diaminobenzidine chromogen. BrdU-positive nuclei were quantified independently by two blinded histologists across ten randomly selected fields per spheroid. Results were expressed as the percentage of BrdU-positive nuclei relative to total nuclei. Only nuclei exhibiting clear, above-background DAB staining were scored as positive.

### 2.5. Monolayer Proliferation Assay

For two-dimensional proliferation analysis, Ishikawa cells were seeded at a density of 3 × 10^5^ cells per well and exposed to LiCl for 24 or 72 h. Following treatment, cells were harvested and counted using a hemocytometer. This assay was performed exclusively in monolayer cultures due to technical limitations associated with dissociation and accurate cell enumeration from spheroids. Experiments were conducted using three independent biological replicates with technical duplicates.

### 2.6. Colony Formation Assay

Clonogenic capacity was evaluated using a double-layer soft agar system. A base layer of 0.6% agar was prepared, followed by a top layer consisting of 0.3% agar containing 3000 viable cells and LiCl at final concentrations of 1, 10, or 50 mM. Plates were incubated for approximately 5 days, with slight variation depending on colony growth kinetics. Colonies containing at least 30 cells were counted under light microscopy. Colony formation efficiency was calculated as the number of colonies formed per 3000 seeded cells.

### 2.7. Transmission Electron Microscopy (TEM)

For ultrastructural evaluation, at least three spheroids per experimental condition were fixed in 2.5% glutaraldehyde and post-fixed with 1% osmium tetroxide. Samples were dehydrated through a graded acetone series and embedded in epoxy resin. Ultrathin sections (~70 nm) were prepared using an ultramicrotome, mounted on copper grids, and contrasted with uranyl acetate and lead citrate. Sections were examined using a JEOL JEM-1011 transmission electron microscope (JEOL Ltd., Tokyo, Japan) operating at 80 kV. Multiple sections and fields were analyzed per spheroid to ensure reproducibility of ultrastructural observations.

For each experimental condition, at least three independent spheroids were processed and multiple non-overlapping fields were examined per ultrathin section for qualitative ultrastructural assessment. Ultrastructural evaluation primarily focused on identifying consistent morphological patterns across different regions of the spheroid. Mitochondrial injury was defined based on established ultrastructural criteria including cristae disruption, matrix swelling, and loss of inner membrane organization.

### 2.8. Statistical Analysis

Statistical analyses were performed using GraphPad Prism version 9.0 (GraphPad Software, San Diego, CA, USA). Data are presented as mean ± standard deviation. Normality was evaluated using the Shapiro–Wilk test. Comparisons between two groups were conducted using Student’s *t*-test when appropriate, while experiments involving multiple variables were analyzed by two-way ANOVA followed by Tukey’s post hoc test. Adjusted *p* values < 0.05 were considered statistically significant. All experiments were performed with three biological replicates unless otherwise stated, and no data points were excluded from analysis.

#### 2.8.1. Use of Generative Artificial Intelligence

Generative artificial intelligence (GenAI) tools were not used in the design of the study, data collection, data analysis, data interpretation, or preparation of the manuscript.

#### 2.8.2. Ethical Approval

This study did not involve human participants or animal experiments; therefore, ethical approval was not required.

## 3. Results

### 3.1. LiCl Alters Spheroid Morphology and Viability

**Figure 1:**
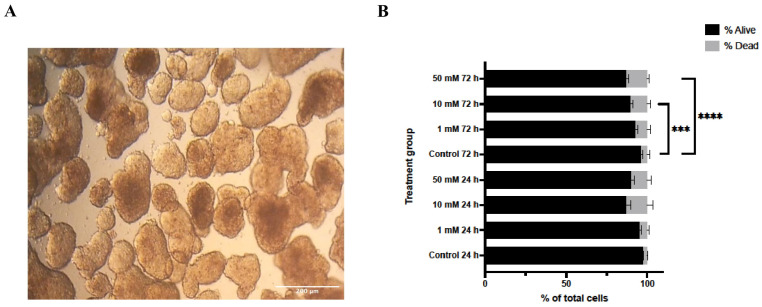
Effects of lithium chloride on spheroid morphology and cell viability. (**A**) Representative bright-field images of 3D Ishikawa spheroids (10×). Control spheroids show compact and well-defined architecture, whereas LiCl exposure results in progressive structural disruption in a dose-dependent manner. (**B**) Trypan Blue exclusion assay quantifying viable and non-viable cells after LiCl treatment. Data are presented as mean ± SD (n = 3). Statistical significance: *** *p* < 0.001, **** *p* < 0.0001.

Lithium chloride (LiCl) exposure induced marked, dose- and time-dependent alterations in the structural integrity of 3D Ishikawa endometrial cancer spheroids. As shown in Figure 1A, untreated control spheroids maintained a compact, spherical morphology with smooth contours throughout the experimental period. In contrast, LiCl-treated spheroids exhibited progressive disruption of spheroid architecture, characterized by loss of compactness, surface irregularities, and diminished structural cohesion. These changes became more pronounced with increasing LiCl concentration and longer exposure duration, with the most extensive disruption observed in spheroids treated with 50 mM LiCl at both 24 and 72 h.

Consistent with these morphological observations, Trypan Blue exclusion analysis demonstrated a concentration- and time-dependent reduction in cell viability following LiCl treatment (Figure 1B). At 24 h, a significant increase in non-viable cells was detected exclusively in the 50 mM LiCl group compared with untreated controls (*p* < 0.0001), whereas 1 mM and 10 mM treatments did not significantly affect viability. After 72 h, both 10 mM (*p* < 0.001) and 50 mM (*p* < 0.0001) LiCl exposures resulted in a marked decline in viable cell numbers, with the highest concentration producing the most pronounced effect.

### 3.2. Lithium Chloride Suppresses Proliferative Activity in 3D Spheroids

**Figure 2:**
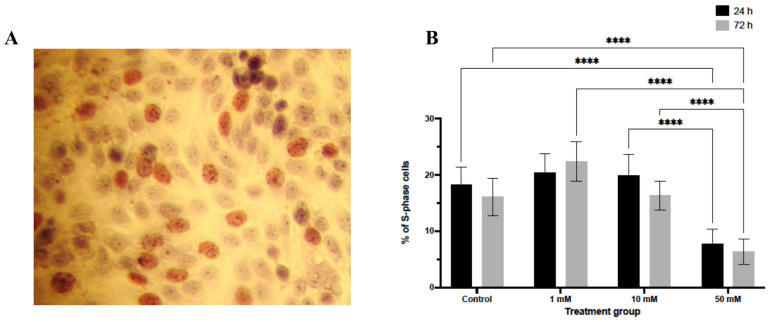
BrdU incorporation analysis in LiCl-treated 3D endometrial cancer spheroids. Ishikawa spheroids were treated with 1, 10, or 50 mM LiCl for 24 or 72 h and stained for BrdU to identify cells undergoing DNA synthesis. BrdU-positive nuclei appear brown in paraffin sections. Quantification was performed by counting labeled and unlabeled nuclei in randomly selected fields (10 randomly sections per group). Data are presented as mean ± SD. Statistical significance: **** *p* < 0.0001. (**A**) Representative immunohistochemical image showing BrdU-positive nuclei in spheroid sections. (**B**) Quantification of BrdU-positive nuclei expressed as a percentage of total nuclei.

Analysis of DNA synthesis by BrdU incorporation revealed a significant reduction in S-phase entry following LiCl exposure (Figure 2). At 24 h, a decrease in the proportion of BrdU-positive nuclei was observed only in spheroids treated with 50 mM LiCl (*p* < 0.0001). After 72 h, both 10 mM and 50 mM LiCl treatments were associated with a substantial reduction in BrdU incorporation (*p* < 0.0001 for both), whereas no significant change was detected at 1 mM. These findings indicate a dose- and time-dependent suppression of proliferative activity in LiCl-treated 3D spheroids.

**Figure 3:**
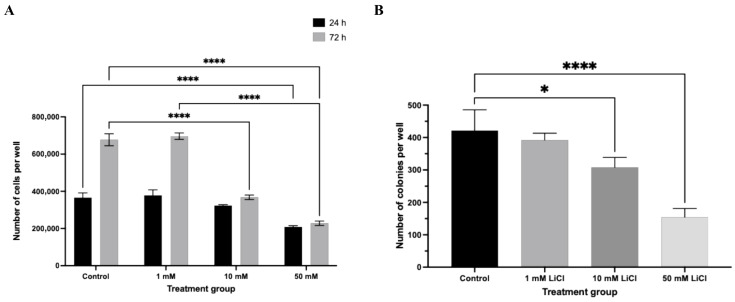
LiCl suppresses total cell number and clonogenic potential in Ishikawa spheroids. (**A**) Following LiCl treatment (1, 10, or 50 mM; 24 or 72 h), spheroids were dissociated and total cell number was quantified using a hemocytometer. (**B**) Clonogenic capacity was evaluated using a soft agar assay. Colonies containing ≥30 cells were counted after 6 days. Data are presented as mean ± SD from three independent experiments. Statistical significance: * *p* < 0.05, **** *p* < 0.0001.

In parallel, total cell number was significantly reduced in a concentration- and duration-dependent manner (Figure 3A). At 24 h, only spheroids treated with 50 mM LiCl showed a significant decrease in total cell count relative to controls (*p* < 0.0001). Following 72 h of exposure, both 10 mM and 50 mM LiCl treatments resulted in a pronounced reduction in total cell number (*p* < 0.0001), whereas spheroids exposed to 1 mM LiCl remained comparable to untreated controls at both time points. These data indicate that spheroid expansion is primarily impaired at higher lithium concentrations and prolonged exposure.

### 3.3. LiCl Reduces Total Cell Number and Clonogenic Capacity

Clonogenic assays further demonstrated that LiCl exposure compromises long-term proliferative capacity in a dose-dependent manner (Figure 3B). Even low-dose LiCl treatment (1 mM) resulted in a modest but statistically significant reduction in colony number (*p* < 0.05). More pronounced suppression of colony formation efficiency was observed following exposure to 10 mM and 50 mM LiCl (*p* < 0.0001), indicating a sustained impairment in the ability of Ishikawa cells to re-initiate proliferative growth following lithium exposure.

### 3.4. Ultrastructural Mitochondrial Alterations in LiCl-Treated Spheroids

**Figure 4:**
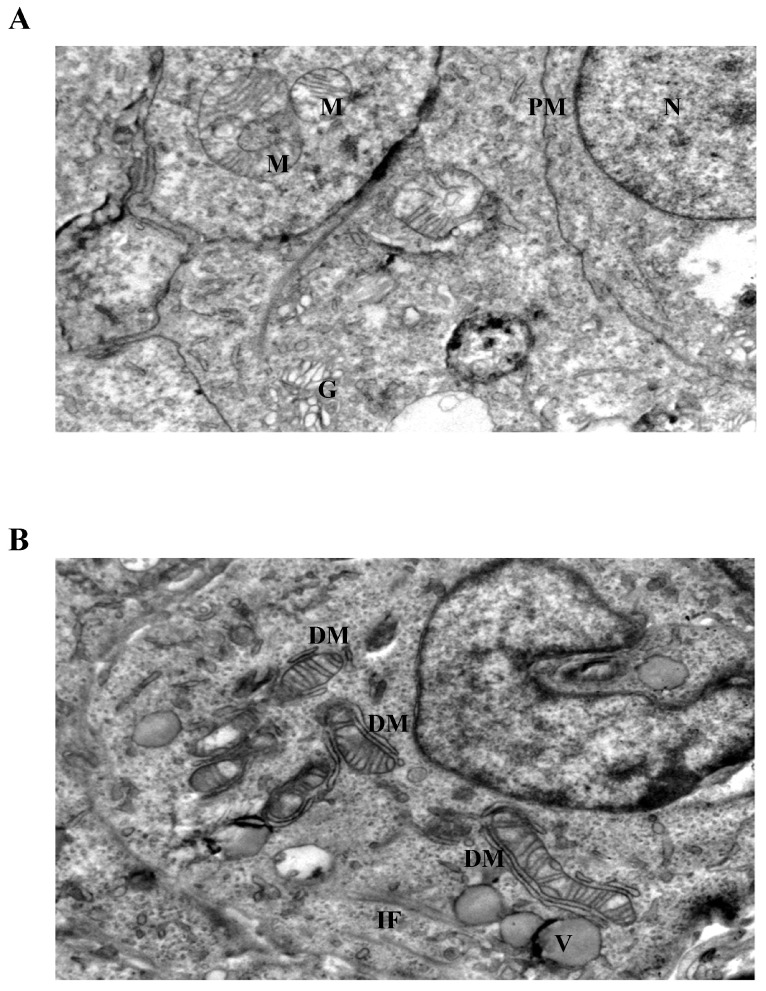
Ultrastructural changes in Ishikawa spheroids following low-dose LiCl exposure (24 h). (**A**) Control spheroids display preserved mitochondrial morphology with intact cristae, organized cytoplasm, and normal plasma membrane integrity. (**B**) Treatment with 1 mM LiCl reveals early mitochondrial alterations, including focal cristae irregularities and double-membrane (DM) isolation structures, accompanied by mild cytoplasmic vacuolization and localized intermediate filament (IF) accumulation. Abbreviations: M, mitochondrion; N, nucleus; G, Golgi apparatus; PM, plasma membrane; DM, double membrane; IF, intermediate filament; V, vacuole; Scale bar: 500 nm.

**Figure 5:**
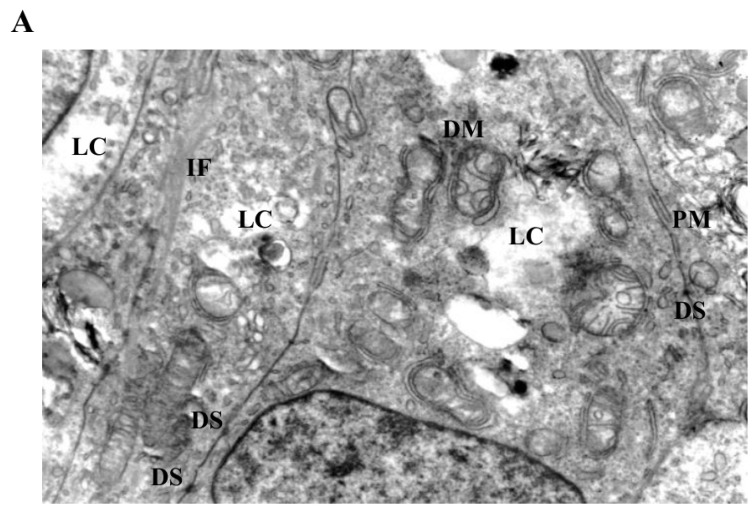

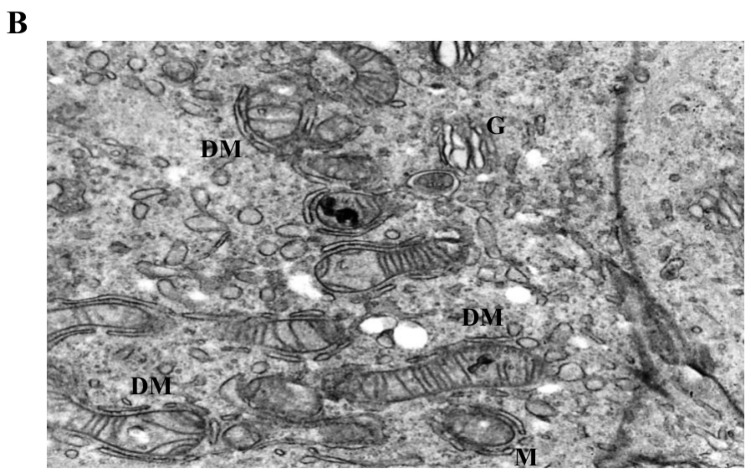
Mitochondrial and cytoskeletal alterations following moderate LiCl exposure. (**A**) At 10 mM LiCl (24 h), mitochondria exhibit pronounced cristae loss, swelling, and frequent DM structures, accompanied by cytoplasmic lysis (LC) and marked IF accumulation. Desmosomal junctions (DS) remain preserved. (**B**) At 1 mM LiCl (72 h), mitochondria display early structural injury with initial cristae disruption and DM formation, consistent with early-stage mitochondrial stress responses. Abbreviations: M, mitochondrion; G, Golgi apparatus; PM, plasma membrane; DM, double membrane; IF, intermediate filament; DS, desmosome; LC, lytic cytoplasm. Scale bar: 500 nm.

**Figure 6:**
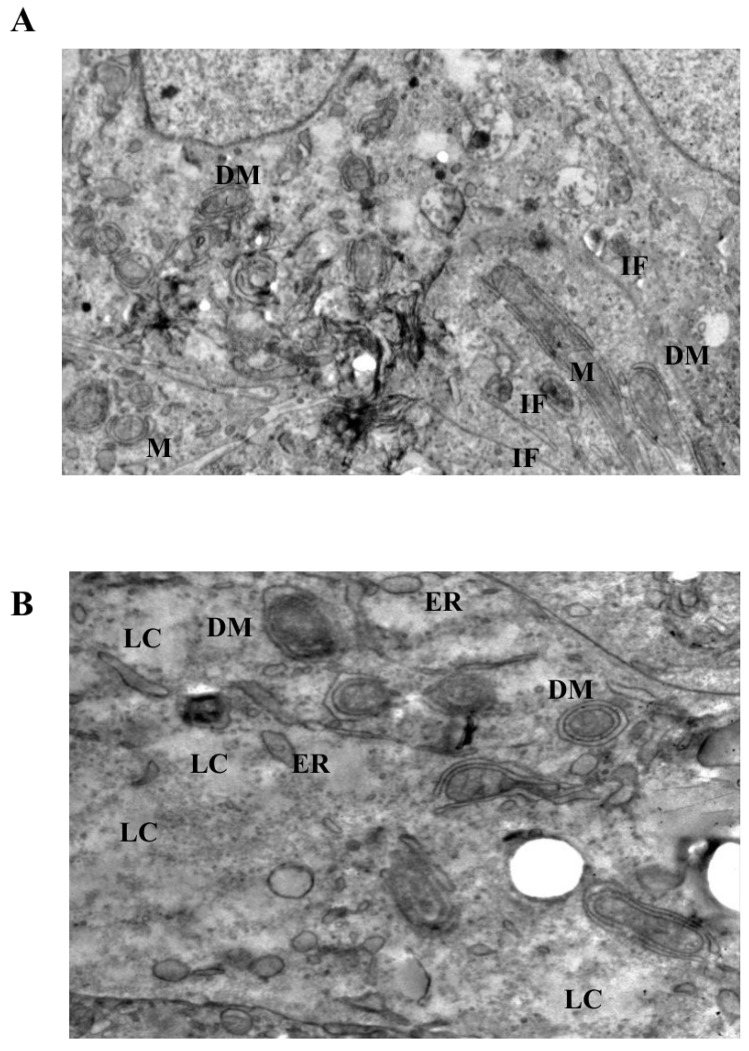
Severe mitochondrial and cytoskeletal disruption induced by prolonged or high-dose LiCl exposure. (**A**) At 10 mM LiCl (72 h), mitochondria show extensive cristae disruption, dense IF accumulation, and increased cytoplasmic lysis, indicating enhanced organelle-centered stress. (**B**) Treatment with 50 mM LiCl results in profound mitochondrial swelling, near-complete cristae loss, and incomplete DM structures, accompanied by dilated endoplasmic reticulum (ER), IF disorganization, and widespread cytoplasmic lysis. Plasma membrane integrity remains largely preserved. Abbreviations: M, mitochondrion; DM, double membrane; IF, intermediate filament; ER, endoplasmic reticulum; LC, lytic cytoplasm. Scale bar: 500 nm.

Ultrastructural analysis by transmission electron microscopy revealed distinct LiCl-induced alterations affecting mitochondrial morphology and intermediate filament organization in 3D Ishikawa endometrial cancer spheroids (Figure 4, Figure 5 and Figure 6). Control spheroids displayed preserved cellular architecture, including mitochondria with intact outer and inner membranes, well-organized cristae, and homogeneous matrix density. The cytoplasm exhibited an organized intermediate filament network, intact plasma membranes, and preserved intercellular junctions (Figure 4A).

Exposure to low-dose LiCl (1 mM) was associated with early mitochondrial alterations, including focal cristae irregularities and the appearance of double-membrane-like structures morphologically resembling isolation membranes in close proximity to mitochondria (Figure 4B). These changes occurred without widespread cytoplasmic lysis, plasma membrane disruption, or global structural disorganization. At 72 h, low-dose LiCl treatment was characterized by an increased frequency of double-membrane structures and subtle mitochondrial matrix alterations (Figure 5B), consistent with an early-stage mitochondrial stress response.

At moderate LiCl concentration (10 mM), mitochondria exhibited more pronounced ultrastructural abnormalities, including cristae depletion, mitochondrial swelling, and frequent double-membrane structures (Figure 5A). Notably, these mitochondrial changes were accompanied by conspicuous accumulation and reorganization of intermediate filaments within the perimitochondrial cytoplasm. Despite these intracellular alterations, plasma membrane integrity and desmosomal junctions remained largely preserved. Prolonged exposure (72 h) further intensified mitochondrial disruption, intermediate filament accumulation, and the appearance of cytoplasmic lytic regions (Figure 6A).

### 3.5. Intermediate Filament Accumulation Accompanies Mitochondrial Injury

Cells exposed to high-dose LiCl (50 mM) displayed the most extensive ultrastructural damage (Figure 6B). Mitochondria showed near-complete cristae loss, marked swelling, and severe disorganization of matrix architecture. Although double-membrane structures were present, their incomplete morphology suggested disrupted or inefficient isolation processes rather than effective organelle clearance. In addition to mitochondrial injury, prominent dilation of endoplasmic reticulum cisternae, diffuse cytoplasmic lysis, and pronounced intermediate filament disorganization were observed. Importantly, across all concentrations and time points examined, classical apoptotic ultrastructural features, such as chromatin condensation, nuclear fragmentation, or apoptotic body formation, were not detected, despite extensive organelle-level alterations.

## 4. Discussion

In this study, we examined the ultrastructural consequences of lithium chloride (LiCl) exposure in 3D Ishikawa endometrial cancer spheroids and identified a cellular stress phenotype characterized by mitochondrial injury and prominent accumulation of intermediate filaments (IFs). These findings indicate that IF networks undergo substantial remodeling under lithium-induced stress and support the notion that cytoskeletal reorganization accompanies mitochondrial dysfunction within a three-dimensional tumor context.

It should be noted that the highest LiCl concentration used in this study (50 mM) exceeds clinically achievable serum lithium levels [34]. In the present experimental framework, this concentration was used to model pharmacological stress conditions rather than therapeutic lithium exposure. Similar supraphysiological lithium concentrations have been applied in several in vitro studies to reveal mitochondrial stress responses and structural cellular adaptations [35]. Therefore, the severe ultrastructural alterations observed at this concentration should be interpreted primarily as indicators of cellular vulnerability under intense pharmacological stress rather than direct representations of clinically relevant lithium exposure. Across multiple experimental endpoints, LiCl exposure exerted clear antiproliferative effects, including reduced cell viability, impaired clonogenic capacity, and decreased BrdU incorporation, particularly at concentrations of 10 mM and 50 mM. These observations are consistent with previous reports demonstrating that LiCl can disrupt proliferative signaling and cellular homeostasis in cancer models [33]. However, as neither GSK3β activity nor apoptosis-associated molecular markers were directly assessed [17,18], the present study does not assign these effects to a specific signaling pathway. Importantly, despite marked reductions in spheroid viability and proliferative capacity, classical ultrastructural hallmarks of apoptosis, such as chromatin condensation, nuclear fragmentation, or apoptotic body formation, were not observed. This pattern suggests that LiCl-induced growth suppression occurs predominantly within a non-apoptotic cellular stress framework rather than through canonical apoptotic execution. However, the absence of classical apoptotic morphology cannot definitively exclude the presence of molecular apoptotic signaling. Additional assays such as caspase activation, Annexin V staining, or TUNEL assays would be required to determine whether apoptotic pathways are partially activated under these conditions. Although lithium is widely recognized as an inhibitor of GSK-3β signaling, the present study did not directly assess pathway activity [35]. Therefore, the relationship between the observed structural alterations and GSK-3β modulation remains speculative and warrants further molecular investigation.

Ultrastructural analyses provided further insight into the nature of this stress response. LiCl-treated spheroids displayed mitochondria with cristae disruption, swelling, and altered matrix organization, frequently accompanied by membrane-associated structures in their vicinity. Such morphological features are commonly interpreted as indicators of mitochondrial stress and engagement of quality-control or adaptive responses, rather than definitive evidence of effective organelle clearance. The presence of these changes even at low LiCl concentrations (1 mM) suggests that lithium can elicit early mitochondrial remodeling under sub-cytotoxic conditions, in agreement with prior ultrastructural studies in cancer models [17,18,33,36,37,38]. At higher concentrations, the progression toward extensive cristae loss and mitochondrial swelling likely reflects a breakdown of adaptive capacity and escalating organelle dysfunction. Because the present work was designed primarily as a descriptive ultrastructural study, mitochondrial alterations and intermediate filament redistribution were evaluated qualitatively rather than through systematic morphometric quantification. Future studies employing digital morphometric analysis or stereological approaches may provide quantitative measurements of mitochondrial injury frequency, cristae density alterations, and intermediate filament distribution across multiple fields.

A particularly notable observation in this study is the consistent accumulation and reorganization of intermediate filaments in close spatial association with stressed mitochondria, most prominently at 10 mM and 50 mM LiCl. This perimitochondrial enrichment of IFs suggests that cytoskeletal remodeling is not merely a secondary consequence of cellular damage but may represent a structural component of the stress response. Intermediate filament proteins are increasingly recognized as dynamic regulators of intracellular organization, signaling, and mechanical stability, and have been implicated in cellular adaptation to metabolic and therapeutic stress [10,11,12,13,27,31,32,39,40]. The spatial convergence of mitochondrial injury and IF accumulation observed here supports the concept of spatial association between organelle stress and cytoskeletal architecture. In this context, intermediate filament remodeling may be interpreted as part of a broader adaptive restructuring process that accompanies mitochondrial stress rather than as a passive byproduct of cellular injury. Because the present study was designed as an ultrastructural investigation, intermediate filament accumulation was evaluated qualitatively rather than through quantitative morphometric analysis. Future studies employing immunofluorescence-based cytoskeletal mapping or digital image analysis will be necessary to quantify filament density and spatial distribution with greater precision.

Dose-dependent differences further underscore this relationship. While low-dose LiCl primarily induced subtle mitochondrial alterations with limited IF rearrangement, exposure to 10 mM LiCl produced pronounced mitochondrial injury together with dense IF accumulation, suggesting a spatial association between mitochondrial injury and intermediate filament remodeling in LiCl-treated spheroids. At 50 mM, extensive mitochondrial disorganization, dilation of endoplasmic reticulum cisternae, cytoplasmic lytic changes, and marked IF network disruption were observed. Notably, despite severe intracellular alterations, plasma membrane integrity and desmosomal junctions were largely preserved, indicating that LiCl-induced injury may remain compartmentalized at the organelle and cytoskeletal levels before loss of overall membrane integrity. Comparable compartmentalized stress patterns have been described in models of severe mitochondrial dysfunction, in which organelle damage precedes overt membrane rupture [7,41]. Endoplasmic reticulum dilation observed at higher lithium concentrations may reflect severe cellular stress and disruption of intracellular homeostasis, phenomena that have been reported in various models of pharmacologically induced mitochondrial dysfunction [42].

Although IF accumulation was a consistent feature across LiCl-treated spheroids, the present study does not distinguish between specific intermediate filament subtypes. Consequently, whether vimentin, keratin 17/19, nestin, or other IF systems selectively contribute to the observed phenotype cannot be determined. Likewise, molecular markers of mitochondrial quality control or autophagy-related pathways were not assessed. Therefore, the findings should be interpreted as ultrastructural evidence of cytoskeletal remodeling associated with organelle-centered stress, rather than as mechanistic proof of defined degradation or cell death pathways. Future studies integrating immunofluorescence, immunoblotting, transcript-level analyses, and functional perturbation of IF networks will be essential to clarify whether IF accumulation reflects adaptive remodeling, impaired turnover, or active participation in stress signaling [10,11,12,13,30,31,32,39].

**Figure 7:**
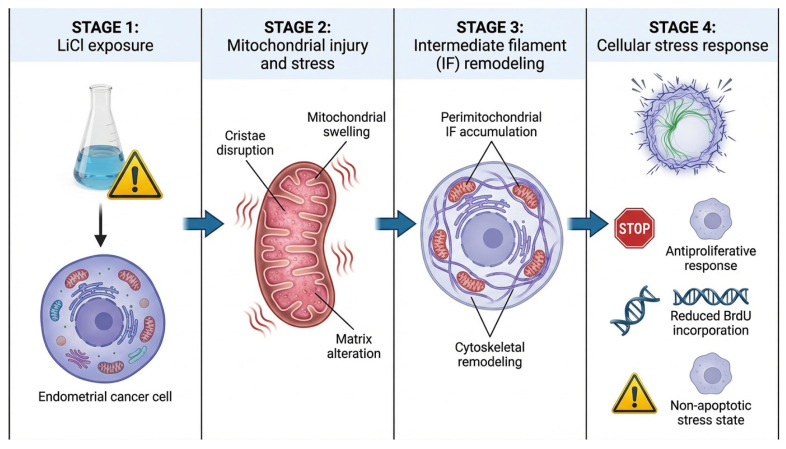
Proposed model of LiCl-induced mitochondrial–cytoskeletal stress response in 3D Ishikawa spheroids. LiCl exposure induces mitochondrial injury characterized by cristae disruption, mitochondrial swelling, and matrix alterations. These mitochondrial changes are accompanied by perimitochondrial accumulation and remodeling of intermediate filaments (IFs), suggesting coordinated cytoskeletal adaptation during organelle-centered cellular stress. The resulting cellular state is associated with antiproliferative effects, reduced BrdU incorporation, and a predominantly non-apoptotic stress response. The schematic illustration was created using graphic illustration tools.

Collectively, these observations indicate that LiCl induces a complex cellular stress state in which mitochondrial dysfunction and cytoskeletal reorganization emerge concurrently. The prominent involvement of intermediate filaments highlights their potential role as integrators of structural and metabolic stress, emphasizing mitochondrial–cytoskeletal relationships as an underexplored dimension of lithium-induced cellular responses in endometrial cancer models and a promising direction for future investigation. Another limitation of the present study is the use of a single endometrial cancer cell line. Although Ishikawa cells represent a widely used model for endometrial adenocarcinoma, validation of these observations in additional endometrial cancer models will be important to determine whether the observed mitochondrial–cytoskeletal phenotype may represent a broader feature of endometrial tumor biology [2,43]. While the present work focuses on ultrastructural changes within three-dimensional spheroids, comparative analysis between 2D monolayer cultures and 3D tumor architectures may further clarify whether mitochondrial–cytoskeletal remodeling is specifically influenced by the structural context of the tumor microenvironment. A schematic summary of the proposed mitochondrial–cytoskeletal stress response is presented in Figure 7.

## 5. Conclusions

In summary, this study provides a detailed ultrastructural characterization of lithium chloride-induced cellular stress in three-dimensional endometrial cancer spheroids. Using transmission electron microscopy in combination with phenotypic assays, we demonstrate that LiCl exposure is associated with pronounced mitochondrial alterations and intermediate filament remodeling in the absence of classical apoptotic morphology. While the present conclusions are derived from ultrastructural observations rather than molecular interrogation, they reveal an organelle-associated organelle-centered and cytoskeletal stress response that extends beyond mitochondrial injury alone. By identifying intermediate filament accumulation as a prominent structural feature of lithium-induced stress in a 3D tumor model, this work establishes a framework for future mechanistic studies aimed at elucidating mitochondrial–cytoskeletal relationships and their relevance to stress adaptation in endometrial cancer.

## Data Availability

The raw data supporting the conclusions of this article will be made available by the authors on request.

## Abbreviations

The following abbreviations are used in this manuscript:

LiCl	Lithium chloride
IF	Intermediate filament
TEM	Transmission electron microscopy
